# What is a recurrence? The onset, frequency and time loss impact of recurrent calf muscle strain injuries in elite male Australian football players over a decade

**DOI:** 10.1136/bmjsem-2025-002865

**Published:** 2025-09-03

**Authors:** Brady Green, Anthony G Schache, Tania Pizzari

**Affiliations:** 1 School of Health Sciences, The University of Notre Dame Australia, Fremantle, Western Australia, Australia; 2 School of Allied Health, Human Services and Sport, La Trobe University, Melbourne, Victoria, Australia; 3 La Trobe Sports and Exercise Medicine Research Centre, La Trobe University, Melbourne, Victoria, Australia

**Keywords:** Recovery, Sporting injuries, Rehabilitation

## Abstract

**Objectives:**

To evaluate the onset, frequency and time loss impact of recurrent calf muscle strain injuries in elite male Australian football players over a decade. To explore how outcomes are affected by alternative recurrence definitions.

**Method:**

Calf muscle strain injuries were reported to the Soft Tissue Injury Registry of the Australian Football League (2014–2023). Cases were categorised as index versus recurrent injuries. Alternate recurrence definitions varied based on: (1) timing (ie, subsequent injuries occurring before or after full recovery (a return to full training) and (2) location (ie, subsequent injuries involving the same side but not necessarily same muscle vs only those confirmed to involve the same muscle).

**Results:**

563 injuries in 359 players were evaluated. Recurrences resulted in ≥2153 total days lost over 10 years and ≥35.6 days lost on average per injury. Recurrence frequencies within 2 years (13%–21.3%), within the same season (7.5%–13.9%) and within 2 months (2.9%–7.3%) varied depending on the definition. 20% of all subsequent injuries occurred before full recovery, and these injuries took on average 46.7±25.6 days to return to play.

**Conclusions:**

Recurrent calf muscle strain injuries in elite male Australian football players commonly have prolonged time loss, irrespective of timing or location. A 2-year recurrence susceptibility is consistent across onsets, and cases that fail early can have a large impact when accounted for. We need to ‘talk the same language’ in research and practice to better understand and prevent recurrences for a given type of injury across different sports and sporting levels.

**WHAT IS ALREADY KNOWN ON THIS TOPIC**

- Recurrent calf muscle strain injuries are problematic in a range of sports, and affect elite and recreational sportspeople.

**WHAT THIS STUDY ADDS**

- While the International Olympic Committee recommendations are available, epidemiological approaches to define recurrence can vary and impact outcomes associated with recurrent calf muscle strain injuries.

**HOW THIS STUDY MIGHT AFFECT RESEARCH, PRACTICE OR POLICY**

- The need for implementation of consensus definitions of recurrence is clear. Further work in this area may also add practical value for interpreting injury before full recovery. This will enable clinicians and researchers involved in different sports to better compare/compile data, which will improve our understanding of the extent of the problem and may contribute to better long-term outcomes.

## Introduction

 Recurrent calf muscle strain injuries are challenging for clinicians, as they are relatively common^1 2^ and often result in prolonged time loss.^1 3^ Knowledge of the epidemiology of recurrent calf muscle strain injuries in elite athletes is limited, considering the magnitude of the problem they represent, and significant variation exists in the literature in key areas, such as diagnosis, subsequent injury definitions and surveillance periods.^24^,^6^ Further evaluation of key recurrence outcomes (eg, the onset, frequency and time loss impact) is required to better understand the aetiology and management, as has been done for injuries affecting other regions, such as the hamstrings.^7 8^

Subsequent injury categorisation in sport is a cornerstone of contemporary epidemiological research^9^,^11^ and consensus recommendations are available to guide researchers and practitioners.^9^ The International Olympic Committee (IOC) has recommended that a recurrence be defined as a subsequent injury occurring after full recovery and affecting the same location and tissue as the index (ie, primary) injury. In instances where the subsequent injury occurs before full recovery (ie, during rehabilitation), it is defined as an exacerbation.^9^ Despite the IOC recommendations becoming available, inconsistency in research methods remains common.

Variation in epidemiological approaches may suggest that the implementation and clinical interpretation of subsequent injury categorisation remain challenging or can lack clarity in the context of research and practical settings.^12^,^15^ One example is the diagnostic threshold required for a muscle strain injury to be defined as a recurrence (eg, is imaging mandatory? Are recurrence outcomes influenced by the use of imaging/radiological diagnosis?). Another issue is whether a subsequent injury to the same complex (eg, calf) but involving a different muscle (eg, soleus vs gastrocnemius) should be considered a recurrence. From a functional perspective, defining consecutive injuries to synergistic muscles within the same complex as unrelated is hard to reconcile clinically. On the other hand, defining these presentations as recurrent would contradict IOC recommendations and may be considered inappropriate, given the details typically used to comprehensively classify a muscle injury (eg, muscle, tissue, anatomical location, position, severity, etc).^16 17^ Finally, despite the IOC recommendations, there is variability in the literature regarding how substantial muscle reinjuries before full recovery are classified (with some studies including these as recurrent,^7 12 14 18^ while others specifically classify them as an exacerbation^19 20^ or a ‘prolongation’ of the index injury^13^). It can be challenging in professional environments to distinguish between subsequent injuries and ‘recurrences’ before full recovery, especially if the athlete needs to restart their rehabilitation process again.^21^ Further research documenting how all these factors influence recurrence outcomes (if at all) is warranted. Exploring the impact of subsequent injury categorisation on recurrence outcomes may provide practical insights for clinicians and help guide future recommendations, alongside key foundational work in this area, such as the IOC recommendations.^9 10 22^

No study has used 10-year, real-world data on muscle strain injuries in elite men’s sport to quantify the extent to which recurrence outcomes change with alternative epidemiological methods for defining subsequent injuries. The aims of this study were to: (1) evaluate the onset, frequency and time loss impact of recurrent calf muscle strain injuries in elite male Australian football for 10 consecutive seasons; and, (2) explore how subsequent injury categorisations alter recurrence outcomes in terms of onset, frequency and time loss impact during this period.

## Materials and methods

### Study design and participants

Data from all calf muscle strain injuries submitted to the Soft Tissue Injury Registry of the Australian Football League (STRAFL) over 10 consecutive seasons (2014–2023) were evaluated, utilising a maximum available sample.^2 14^ Injuries affected elite Australian football players participating in the professional men’s competition—the Australian Football League.

### Data collection

Time loss calf muscle strain injuries were reported after diagnosis from a team doctor and/or physiotherapist. Physiotherapists used a standardised injury reporting form to submit injuries to the STRAFL (online supplemental file 1), which has been described previously^3 6 18 23^ and includes information about: (1) player demographics; (2) injury onset and (3) recovery outcomes (eg, days to return to full training and return to play (RTP)).^24^ For the current study, data included the date of injury, recovery outcomes and MRI reports to confirm muscle involvement only (which has excellent inter-rater agreement for calf injuries).^17 25^ Where possible, data items and reporting met IOC consensus recommendations (including the STROBE-SIIS).^9^ The STRAFL does not receive quantitative load data or data about uninjured players. Injuries from the preseason and in-season periods are submitted (online supplemental file 1).

### Data handling and standardisation

#### Subsequent injury definitions and recurrence frequency

Players were assigned a unique identification code, and injuries were categorised to identify index and subsequent injuries.^4 9^ An index injury was defined as the first chronologically recorded calf muscle strain injury affecting the leg within a 2-year surveillance period.^9 11^ Future calf muscle strain injuries to the same leg within a maximum surveillance period of 2 years of the date of the index injury were defined as subsequent injuries.^9 11^ Whether a subsequent injury was categorised as a recurrence or not varied based on the epidemiological definition (table 1): The timing of onset (before/after recovery) and whether location-specificity was required (same/different calf muscle) were the adjusted variables between the definitions. Categorisation began using the simplest epidemiological approach (table 1: D1), and progressed in ascending order based on the number of variables to consider (ie, D4 included both variables and was completed last). For approaches relying on an imaging threshold/radiological diagnosis to define recurrence, MRI reports were accessed to verify the location (same muscle) (table 1: D3 and D4). Time to recurrence was defined as the number of days.^9^ It was quantified as the cumulative incidence, and data were also categorised to describe the onset: 0–2 months, 2–6 months, 6–12 months, 12 months–2 years.^7 8 12 15 22 26^

**Table 1:** Epidemiological approaches to define recurrence

Definition	Surveillance	Onset	Location	Recurrence definition
D1	2 years	Any time ≤2 years	Same leg	Recurrences are subsequent calf muscle strain injuries affecting the same leg, occurring at any stage after the index injury within a 2-year period.
D2	2 years	After full recovery, and ≤2 years	Same leg	Recurrences are subsequent calf muscle strain injuries affecting the same leg, occurring after full recovery and within a 2-year period.
D3	2 years	Any time ≤2 years	Same muscle	Recurrences are subsequent calf muscle strain injuries affecting the same muscle as the index injury, occurring at any stage after the index injury within a 2-year period.
D4	2 years	After full recovery, and ≤2 years	Same muscle	Recurrences are subsequent calf muscle strain injuries affecting the same muscle as the index injury, occurring after full recovery but within a 2-year period.

In D2 and D4, records of calf muscle strain injuries occurring before full recovery (ie, during the rehabilitation period) were not defined as recurrences and, therefore, these presentations were not included in the respective counts of new injuries/recurrences.

In D3 and D4, MRI data were required to confirm that the same location (muscle) was affected.

Cases where muscle involvement could not be confirmed were not defined as recurrent.

#### Time loss definitions and impact

Time loss (days) after recurrent calf muscle strain injury was quantified as the number of days to RTP.^9^ In 1.07% of cases, the return-to-full-training date was used instead of RTP because there were no scheduled matches (eg, it was a preseason injury) and the player had fully recovered without subsequent injury.^27^ Severity categories were also used to describe time loss, using a previously researched approach in muscle strain injuries affecting elite athletes: (1) slight: 0 days; (2) minimal: 1–3 days; (3) mild: 4–7 days; (4) moderate: 8–28 days; (5) severe: >28 days.^1 7 12^

### Statistical analyses

#### Recurrence onset and frequency

2-year and same-season recurrence frequencies were calculated for all definitions. Recurrence frequencies before full recovery (ie, during rehabilitation) were included for D1 and D3. For D2 and D4, calf muscle strain injuries that occurred before full recovery were defined as exacerbations (a prolongation of the initial injury) and were not counted as individual cases of calf muscle strain injury or as recurrences^9 13 15^ (table 1). For all definitions (D1–D4), the time to recurrence (in the number of days) was explored using descriptive statistics and categorisation (eg, prevalence of onset: 0–2 months, 2–6 months, 6–12 months, 12 months–2 years).^6 8 18^

#### Time loss impact

Time loss impact was quantified for all definitions as the total number of days lost: (a) over 10 years (2014–23) and (b) per season. Time loss per recurrence was then explored using descriptive statistics. These data were also categorised to describe time loss according to recurrence onset^4 26 28^: early: within 2 months; late: 2–12 months; delayed: >12 months.

Kaplan-Meier survival analysis was used to measure time to RTP for all recurrent calf muscle strain injuries relative to index injury cases (log rank (Mantel-Cox) test and survival curve visualisation), with this comparison helping to describe the impact of recurrences. Cases were censored if the injured player did not RTP during the surveillance period, such as when another injury was sustained or the season ended, which also helped to avoid inflated time loss periods.^29 30^ All data were analysed using SPSS Statistics (V.29.000 (241), IBM), with significance set at p<0.05. Statistical analyses and presentation were guided by and are consistent with the CHAMP statement.^31^

## Results

### Overview

563 calf muscle strain injuries reported to the STRAFL were evaluated. Injuries occurred in 359 players. There were 101 (28.1%) players who sustained ≥2 subsequent injuries of any type and onset, including calf muscle strain injuries to the contralateral leg. Recovery data were available in 76.5% of cases.

### Recurrence onset and frequency

2-year recurrence frequencies ranged between 13.0% and 21.3% (table 2). Over 50% of recurrences occurred ≤6 months after the index injury (table 2). While subsequent injuries within the same season made up the majority (≥56.3%) of all recurrent calf muscle strain injuries, the frequency of same-season recurrences changed from 13.9% to 7.5% when describing the most simple (ie, anytime ≤2 years; same leg) vs the most involved (ie, ≤2 years; same leg; MRI-confirmed same muscle; after full recovery) recurrence definition (table 2). Early recurrences (ie, 0–2 months) were the most prevalent for D1 (34.2%) and D3 (35.9%) (table 2). When subsequent injuries occurring before full recovery were not defined as recurrent, the frequency of early recurrence was approximately half that of approaches that included them (D1 vs D2 or D3 vs D4 in table 2). The median time to recurrence for subsequent injuries occurring before full recovery was 22 days for D1 and 22.5 days for D3, which was around two-thirds (D1: 71%; D3: 64%) of the way through the entire rehabilitation period (ie, the median time to recurrence/the median time to RTP). No recurrences occurred between returning to full training and RTP. Three recurrences specifically occurred in the return match.

**Table 2:** Recurrent calf muscle strain injury frequency in the AFL 2014–2023

	D1 (n=563): ≤2 years, same leg		D2 (n=539): After recovery and ≤2 years, same leg		D3 (n=563): ≤2 years, same muscle		D4 (n=545): After recovery and≤2 years, same muscle	
	n (%)	Proportion (%)	n (%)	Proportion (%)	n (%)	Proportion (%)	n (%)	Proportion (%)
Total 2-year recurrences	120 (21.3)	100.0	96 (17.7)	100.0	89 (15.8)	100.0	71 (13.0)	100.0
Same season recurrences	78 (13.9)	65.0	54 (10.0)	56.3	59 (10.5)	66.3	41 (7.5)	57.7
Recurrences prior to recovery	24 (4.3)	20.0	N/A		18 (3.2)	20.2	N/A	
Time-course of recurrence								
0–2 months	41 (7.3)	34.2	19 (3.5)	19.7	32 (5.7)	35.9	16 (2.9)	22.5
2–6 months	35 (6.2)	29.2	31 (5.8)	32.3	26 (4.6)	29.2	22 (4.0)	30.9
6–12 months	20 (3.6)	16.7	22 (4.1)	22.9	11 (1.9)	12.4	13 (2.4)	18.3
12 months–2 years	24 (4.3)	20.0	24 (4.3)	25.0	20 (3.6)	22.5	20 (3.7)	28.2
Time to recurrence								
Overall (Mdn (IQR), range)	109 (44–315.75), 4–697		172 (70.25–374.45), 15–697		84 (41.5–326.5), 4–697		168(68–385), 15–697	
Prior to recovery (Mdn (IQR), range)	22 (16–28.75), 4–78		N/A		22.5 (19.75–31.5), 4–59		N/A	

Proportion (%): refers to the percentage each recurrence onset represents within the total number of recurrent calf muscle strain injuries.

AFL, Australian Football League; D, definition; Mdn, median; n, number; N/A, not applicable.

### Time loss impact

#### Overall time loss: 10-year and per-season impact

**Figure 1:**
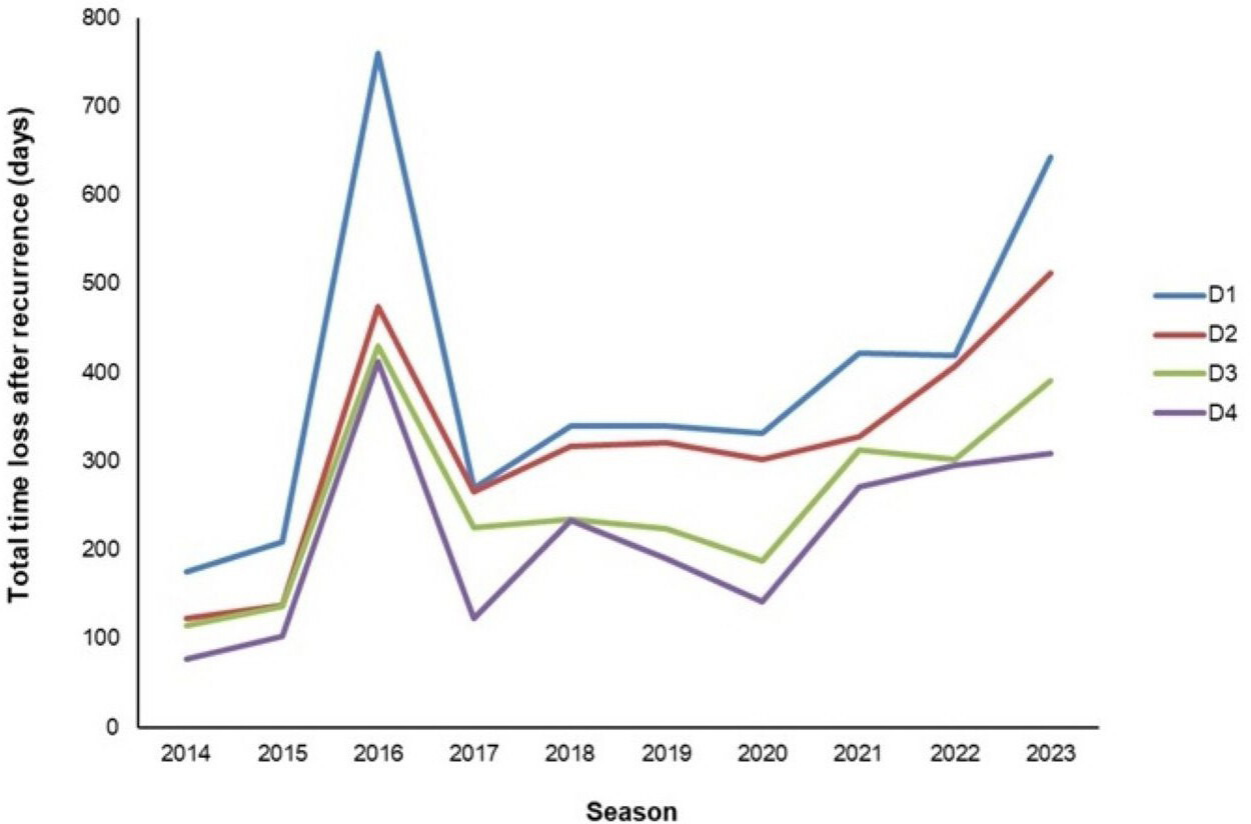
Time loss impact of recurrent calf muscle strain injuries per season. D1=definition1; D2=definition 2; D3=definition 3; D4=definition 4.

Recurrent calf muscle strain injuries resulted in ≥2153 days lost over 10 years (table 3). Between the start and end of the surveillance period (ie, when comparing 2014–2023), the per-season time loss impact of recurrences rose 468 days (D1), 389 days (D2), 275 days (D3) and 231 days (D4) (figure 1 and online supplemental table 2). Seasonal time loss was highest in 2016 (figure 1). After 2016, the average impact of recurrent calf muscle strain injuries ranged between 223 and 395 days lost annually (online supplemental table 2).

**Table 3:** The 10-year time loss (days) impact of recurrent calf muscle strain injuries in the AFL: 2014–2023

	D1: ≤2 years, same leg	D2: After recovery and ≤2 years, same leg	D3: ≤2 years, same muscle	D4: After recovery and ≤2 years, same muscle
Total 10-year time loss	3907	3187	2556	2153
Time loss per injury				
Index injuries	25.1±16.4, 21 (14–33), 2–109	25.2±16.5, 21(20–48), 2–109	25.6±17.1, 21 (14–33), 2–109	25.8±17.4, 21 (14–33.3), 2–109
Recurrences	37.2±23.5, 30 (20–49), 7–102*	36.2±22.6, 30 (20–48), 7–102*	38.7±23.2, 33 (21.3–49), 7–102*	35.6±25.6, 30 (21–47.3), 12–102*
Recurrence onset				
Prior to full recovery	46.7±25.6, 38 (30–57), 12–90	N/A	46.7±25.6, 38 (30–57), 12–90	N/A
Early: ≤2 months	40.3±26.8, 30 (21.5–56), 12-102	36.9±26.8, 28 (20.5–45), 12–102	45.3±28.9, 35 (22.5–68.5), 12–102	37.6±26.1, 27 (20–55), 14–102
Late: 2–12 months	37.4±23.0, 32 (21.5–48.5), 9-101	35.9±22.7, 30.5 (20–47.5), 9–101	37.5±19.5, 28 (24–48), 13–101	40.9±23.3, 35 (26–49), 13–101
Delayed: >12 months	37.4±21.7, 40.5 (18–50.5), 7–88	37.4±21.7, 40.5 (18–50.5), 7–88	35.5±21.9, 39 (17–47), 7–88	35.5±21.9, 39 (17–47), 12–88
Recurrence severity				
Mild: 4–7 days	1 (1.1%)	1 (1.0%)	1 (1.6%)	0 (0.0%)
Moderate: 8–28 days	40 (44.4%)	38 (44.7%)	25 (39.1%)	23 (45.1%)
Severe: >28 days	49 (54.4%)	46 (54.1%)	38 (59.4%)	28 (54.9%)

Recovery is presented as mean±SD, median (IQR: 25th–75th percentile), range.

* Longer recovery time compared to index injury (p<0.05).

AFL, Australian Football League; N/A, not available.

#### Individual time loss: per-injury impact

Recurrences were on average severe (ie, >28 days to RTP), taking (mean±SD) 35.6±25.6 (D4) to 38.7±23.2 (D3) days to recover (online supplemental table 1). Recurrences that specifically occurred before full recovery were, on average, also severe (table 3; online supplemental figure 1). Recurrent injuries took longer time to RTP compared with index injuries irrespective of the definition (D1: X^2^(1)=35.8, p<0.001; D2: X^2^(1)=28.8, p<0.001; D3: X^2^(1)=34.4, p<0.001; D4: X^2^(1)=32.6 p<0.001) (online supplemental tables 2–5 and figures 2–5).

## Discussion

This study presents key recurrence outcomes (onset, frequency, time loss impact) for calf muscle strain injuries in elite male Australian football. It is the first elite-level, data-led exploration of how subsequent injury categorisation definitions affect recurrence outcomes over a decade. Four major findings were revealed: (1) recurrence outcomes depend on the epidemiological method used to define recurrence; (2) recurrences are problematic for a prolonged period; (3) the overall and per-injury impact of recurrent injuries was often severe (median and mean RTP>28 days)^1 7 32^ and (4) subsequent injuries that occur before full recovery impact outcomes when accounted for and warrant consideration.

More than 1-in-5 subsequent calf muscle strain injuries within 2 years may be recurrent (D1). Even if more stringent criteria are employed regarding recurrence definition (ie, MRI-confirmation/radiological diagnosis, and onset occurring after full recovery only), recurrences represent approximately one in eight reported calf muscle strain injuries (eg, D4). Recurrence frequencies demonstrated in the current study reflect and exceed previous data^1 6 32^ and are consistent with hamstring muscle strain injuries.^7^ From this perspective, our findings may highlight the contemporary impact of calf muscle strain injuries in sports.

Muscle-specific susceptibility to recurrence is high for 6 months, representing up to two-thirds of cases. Consistent with our findings, Orchard *et al* demonstrated elite Australian football players remain at an elevated risk of recurrence after a calf muscle strain injury for around 4 months after RTP.^2^ Tissues within the calf muscle-tendon unit can take prolonged periods to remodel and mature following injury. Since muscle strains are unlikely to be fully healed at the time of RTP from a pathoanatomical perspective^33^—compromised structural integrity within the recently injured calf muscle is a logical explanation for the high and persistent rates of early recurrence. Structural changes after a calf muscle strain injury can also persist,^34^ including scarring^3^ and altered architecture,^35^ which may disrupt function and predispose to recurrence. From a performance perspective, greater running workloads imposed on players during games can increase the risk of subsequent calf muscle strain injury.^36^ Since index calf muscle strain injuries incur altered loading to some extent, followed by the resumption and progressive reconditioning of running capacities, athletes may be susceptible to recurrence with a rapid increment in workload either during the end stage of rehabilitation or on RTP.^21^ Definitions that require location-confirmation using MRI (D3, D4) may be best suited to identify cases where exposure likely exceeded the capacity of a recovering muscle. Subsequent injury categorisation to this extent (and potentially further) may only be achievable in elite/well-resourced settings.

Subsequent calf muscle strain injuries that occur before full recovery require specific attention due to the impact they can have on the player and the competition. These presentations accounted for 20% of the recurrences in D1 and D3, resulting in an average of severe time loss. Excluding these presentations can result in approximately 50% fewer early recurrences being recorded when examining D1 versus D2 and D3 versus D4. Based on prevalence and impact, considering these injuries in recurrence prevention appears warranted. Unique to calf muscle strain injuries, experts have previously identified that particular stages within the rehabilitation programme pose an elevated risk—such as when running volume and intensity are progressed.^21^ In the current study, subsequent injuries occurring before full recovery typically occurred around 22 days after the index injury, or 64%–71% of the median recovery length (ie, two-thirds of the way through rehabilitation), which is consistent with these findings. Regardless of how they are epidemiologically handled, the reasons underlying subsequent calf muscle strain injuries that occur before full recovery require attention in future research. Furthermore, lower limb muscle strain injuries may require specialised subsequent injury categorisation approaches due to their unique characteristics compared with other injury types.

The overall impact of recurrent calf muscle strain injuries appears to be higher than it was 10 years ago. Improved surveillance and diagnostic approaches may partly explain this finding. For example, better identification and recognition of injuries involving the soleus,^37^ which are the most prevalent in elite Australian football^6 38^ and may have been underrepresented historically. It is also possible that over a decade, there is a greater proportion of players captured within the STRAFL with risk factors for subsequent calf muscle strain injury.^39^ Running workloads during training and matches may have increased over the decade as well. Despite these possible explanations, the persistent overall impact of recurrent calf muscle strain injuries suggests that strategies to prevent recurrences require further refinement. This may be especially true when considering how consistently impactful recurrences are at the individual athlete level (ie, time lost per injury).

Recurrent calf muscle strain injuries affecting elite Australian football players are usually severe, taking at least 35.6 days to RTP on average (D4). With most matches scheduled within 1 week event cycles, when a recurrence does occur, players may be expected to be unavailable for approximately 4–6 matches on average. Data from elite athletics have similarly shown the substantial impact of calf muscle strain injuries per injury, with soleus injuries representing the third-highest burden over three seasons (only behind hamstring muscle strain injuries and Achilles tendinopathy). Still, the proportion of these that are recurrent (plus analysis relative to onset) is yet to be available.^27^ The high loads placed on the calf muscles, particularly the soleus, during running are well established.^40 41^ From this perspective, prolonged time loss after a recurrence is understandable, especially when recognising that at RTP Australian football players will commonly cover on average >12 000 m total distance, >1000 m high-speed running (≥5.5 m/s) and >150 m sprinting (≥7 m/s), as well as >15 accelerations (≥2.78 m/s²) and >32 decelerations (≤-2.78 m/s²).^42^ Irrespective of pathology severity, it can take time to safely restore these capacities while mitigating the risk of another recurrence.^21^ The implications of suboptimal management and/or risk mitigation strategies may also contribute to the high frequency of early and same-season recurrences.

### Clinical applications

With new insight into the long-term behaviour of key recurrence outcomes (onset, frequency and time loss impact), the priority of recurrent calf muscle strain injuries for secondary and tertiary prevention strategies is clear. Recognising that varied information is available to researchers and practitioners based on the setting (eg, elite vs subelite and recreational), these data may provide insight if routine imaging is not feasible. Where possible, the IOC consensus definition for injury recurrence should be followed to facilitate data comparison across various international sports injury surveillance systems.

### Strengths and limitations

This is the first study to use a decade of surveillance data from an elite sport to explore recurrence outcomes after calf muscle strain injuries. Further research is needed to combine findings from the current study with a larger exploration of recurrences affecting precisely the same anatomical location using MRI. We have completed a preliminary analysis in this area, but this evidence would benefit from a larger sample size.^3^ Due to this surveillance project starting in the 2014 season, with not all teams participating, it may be partly expected that a lower time loss impact would be measured in the first few seasons. Full recovery data were not available in all cases, suggesting the true impact of recurrence is higher. For the duration of the surveillance period (2014–2023), there has not been consistency within the STRAFL (and in other sporting codes) regarding how to define and report subsequent calf muscle strain injuries. As a result, the number of subsequent injuries that occurred before full recovery may have been under-reported. It is also possible that some recurrences did not result in time loss and were not reported.

## Conclusions

The criteria used to define a recurrence impact key outcomes associated with recurrent calf muscle strain injuries. Depending on the definition, up to 1-in-5 cases are recurrent, and almost two-thirds may fall ≤6 months. Information about subsequent injuries may help guide practitioners in a range of settings in management and prevention, including recognising the significant impact when rehabilitation fails early. It should also be acknowledged that even with recommended IOC terminology becoming available, the threshold that represents a reportable injury before full recovery is not always straightforward. On opposing ends of the spectrum are ‘exacerbations’ that completely halt rehabilitation and require the process to begin from ‘square one’ vs scenarios where progress plateaus temporarily due to mild symptoms—but an athlete’s prognosis remains largely unaltered. Not recording these presentations has been reported as a limitation of previously conducted prospective muscle injury research focusing on recurrences.^15^ Further nuance may exist within current recommendations, and considering these subsequent injuries in reporting adds value to understanding the aetiology of recurrence and improving management.

## Supplementary material

10.1136/bmjsem-2025-002865 online supplemental file 1

10.1136/bmjsem-2025-002865 online supplemental file 2

## Data Availability

No data are available.
