# Supplementary material for: What is a recurrence? The onset, frequency and time loss impact of recurrent calf muscle strain injuries in elite male Australian football players over a decade
Source: BMJ Open Sport Exerc Med. 2025 Sep 3;11(3):e002865. doi: 10.1136/bmjsem-2025-002865 (PMC12410626; doi:10.1136/bmjsem-2025-002865)
Supplement: online supplemental file 1 [file bmjsem-11-3-s001.pdf]

Supplementary file 1. Raw STRAFL variables list for calf muscle strain injuries

**Variable**	**Data/ brief description**
Club	• Team the injured player is from
Player surname	• Last name of injured player
Player given name	• First name of injured player
Consent form	• Confirmation of player consent: Yes/No
Radiological scan collected	• Whether imaging was performed: Yes/No
Date of scan	• Date of imaging
Date of injury	• Date of injury diagnosis
Recurrence of injury in the same season	• Yes/ No
If recurrence, provide details	• Free text: E.g. injured sides, dates, outcomes
Playing position	• Forward/ Back line/ Midfielder/ Ruck/ Utility
Ethnicity	• Indigenous/ Non-Indigenous
Previous calf injuries	• Yes/ No
Side of previous calf injuries	• Left/ Right/ Left and right/ N/A
Details previous calf injuries	• Free text to further describe calf strain history
Previous ankle injuries	• Yes/ No
Side of previous ankle injuries	• Left/ Right/ Left and right/ N/A
Details previous ankle injuries	• Free text to further describe ankle injury history
Previous foot injuries	• Yes/ No
Side of previous foot injury	• Left/ Right/ Left and right/ N/A
Details of previous foot injury	• Free text to further describe foot injury history
Uses orthotics	• Yes/ No
Change in footwear prior to injury	• Yes/ No
Change in loading prior to injury	• Yes/ No
Details of change in loading prior to injury	• Free text to further describe change in loading prior to injury
Other injuries	• Free text to further describe other relevant injury history
Activity that the injury occurred	• Training/ Match/ Other
Game details (for match injuries)	• 1st quarter injury/ 2nd quarter injury/ 3rd quarter injury/ 4th quarter injury/ N/A
Percentage of game time played	• Free text: Numeric value

Number of minutes played prior to injury	• Free text: Numeric value
Number of continuous minutes played prior to injury	• Free text: Numeric value
Injury side	• Left (dominant)/ Left (non-dominant)/ Right (dominant)/ Right (non-dominant)
Mechanism of injury	• Acceleration/ High intensity running/ Steady state running/ Sprinting with lumbar flexion/ Deceleration/ Kicking (stance leg)/ Kicking (kicking leg)/ Sudden change of direction/ Jumping/ Landing/ Gradual onset/ Nil-specific
Ground of injury	• Free text to record ground that the injury occurred
Ground week prior	• Free text to record ground played at the week before the injury
Usual training venue	• Free text to record the usual training venue
Completed >80% of the preseason	• Yes/ No
Estimated percentage (%) preseason completed	• Free text: Numeric value
Percentage (%) game time 1-week prior	• Free text: Numeric value
Percentage (%) game time 2-weeks prior	• Free text: Numeric value
Percentage (%) game time 3-weeks prior	• Free text: Numeric value
Time to walk pain free	• Number of days taken to walk pain free after injury
Time to run at >90% of maximum speed	• Number of days taken to run at >90% of maximum speed after injury
Time to return to full training	• Number of days taken to train fully after injury
Time to return to play	• Number of days taken to return to play after injury
Age	• Years
Height	• Centimetres (cm)
Weight/ Body mass	• Kilograms (kg)
Career AFL games	• Number of senior AFL matches played
Years on an AFL list	• Years AFL-listed
