# Supplementary material for: What is a recurrence? The onset, frequency and time loss impact of recurrent calf muscle strain injuries in elite male Australian football players over a decade
Source: BMJ Open Sport Exerc Med. 2025 Sep 3;11(3):e002865. doi: 10.1136/bmjsem-2025-002865 (PMC12410626; doi:10.1136/bmjsem-2025-002865)
Supplement: online supplemental file 2 [file bmjsem-11-3-s002.pdf]

Supplementary file 2

Supplementary figure 1. Time loss impact of recurrent calf muscle strain injuries using D1-D4

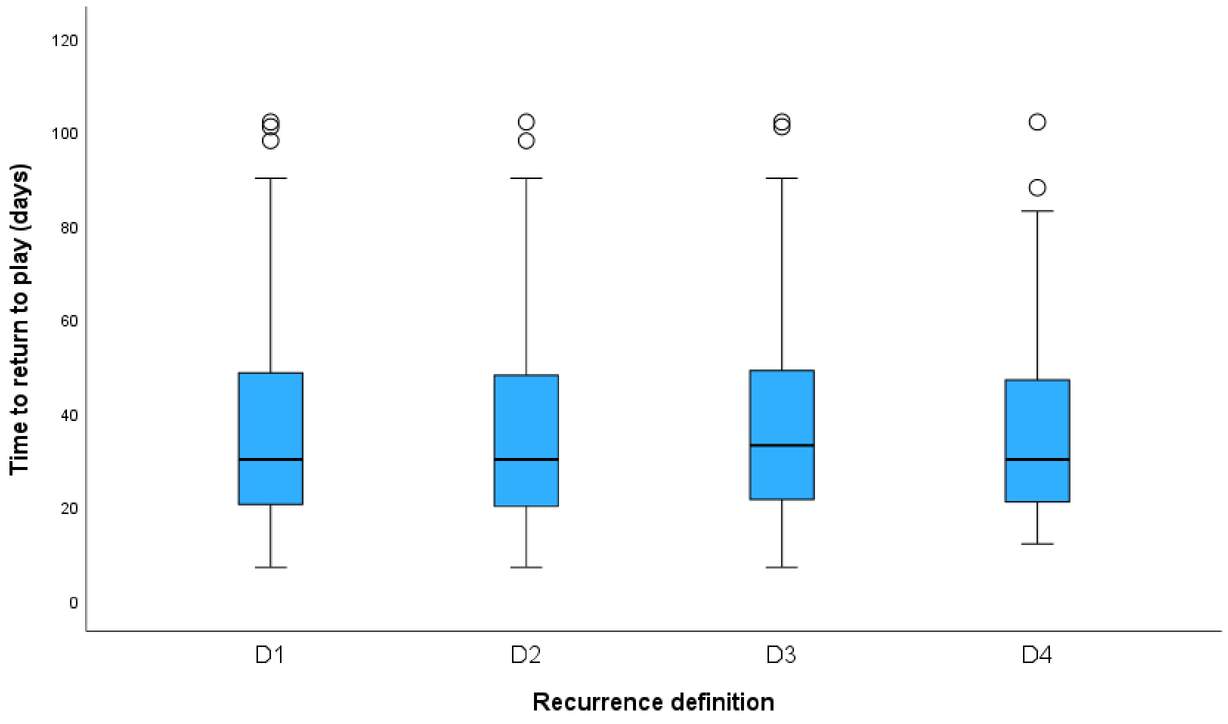

Supplementary table 1. Total time loss impact (days to return to play) after recurrent calf muscle strain injuries using: 2014-23.

	D1 (n=120):<br>≤2 years, same leg	D2 (n=96):<br>After recovery and ≤2 years, same leg	D3 (n=89):<br>≤2 years, same muscle	D4 (n=71):<br>After recovery and ≤2 years, same muscle
**Ten-year time loss**				
2014	175	123	115	77
2015	209	138	136	103
2016	759	474	430	412
2017	270	266	225	122
2018	339	317	235	233
2019	340	320	224	190
2020	331	302	187	142
2021	422	328	312	271
2022	419	407	302	295
2023	643	512	390	308
Total	3907	3187	2556	2153

Supplementary figure 2 & table 2. Outputs from time to event analysis comparing recovery of index and recurrent CMSI using D1.

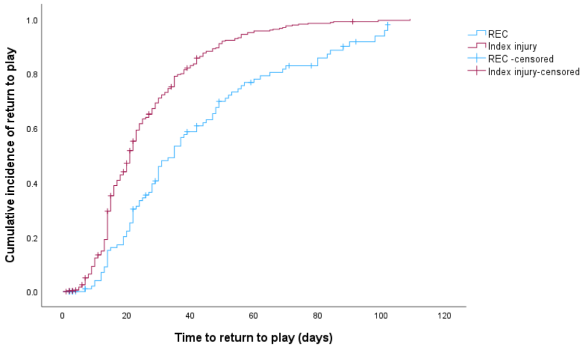

Means and Medians for Survival Time

REC_Vs_Index	Mean				Median			
			95% Confidence Interval				95% Confidence Interval	
	Estimate	Std. Error	Lower Bound	Upper Bound	Estimate	Std. Error	Lower Bound	Upper Bound
REC	42.373	2.826	36.834	47.913	35.000	2.553	29.995	40.005
Index injury	25.902	.932	24.075	27.730	21.000	.712	19.604	22.396
Overall	29.518	1.011	27.537	31.499	23.000	.686	21.655	24.345

Supplementary figure 3 & table 3. Outputs from time to event analysis comparing recovery of index and recurrent CMSI using D2.

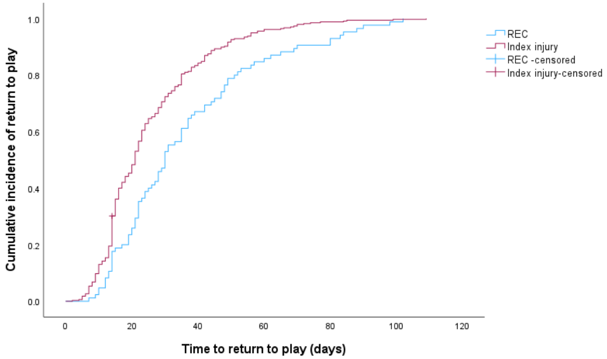

Means and Medians for Survival Time

REC_Vs_Index_Inj	Mean[a]				Median			
			95% Confidence Interval				95% Confidence Interval	
	Estimate	Std. Error	Lower Bound	Upper Bound	Estimate	Std. Error	Lower Bound	Upper Bound
REC injury	36.235	2.452	31.429	41.042	30.000	1.673	26.720	33.280
Index injury	25.199	.900	23.435	26.963	21.000	.871	19.292	22.708
Overall	27.422	.897	25.664	29.180	22.000	.615	20.795	23.205

a. Estimation is limited to the largest survival time if it is censored.

Supplementary figure 4 & table 4. Outputs from time to event analysis comparing recovery of index and recurrent CMSI using D3.

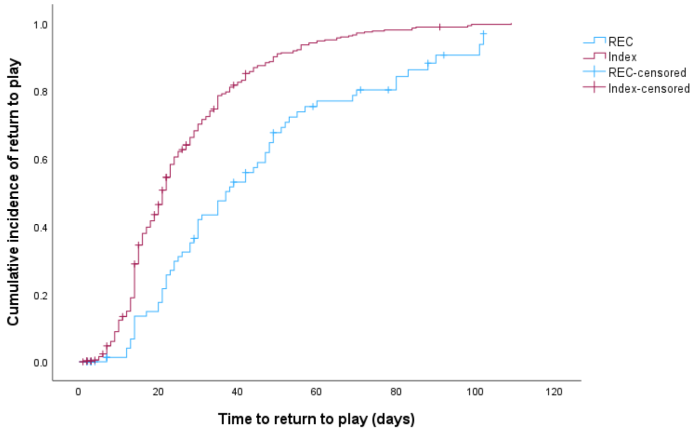

Means and Medians for Survival Time

	Mean				Median			
			95% Confidence Interval				95% Confidence Interval	
REC_Vs_Index	Estimate	Std. Error	Lower Bound	Upper Bound	Estimate	Std. Error	Lower Bound	Upper Bound
REC	45.066	3.329	38.541	51.591	37.000	5.133	26.940	47.060
Index	26.431	.933	24.601	28.261	21.000	.695	19.638	22.362
Overall	29.518	1.011	27.537	31.499	23.000	.686	21.655	24.345

Supplementary figure 5 & table 5. Outputs from time to event analysis comparing recovery of index and recurrent CMSI using D4.

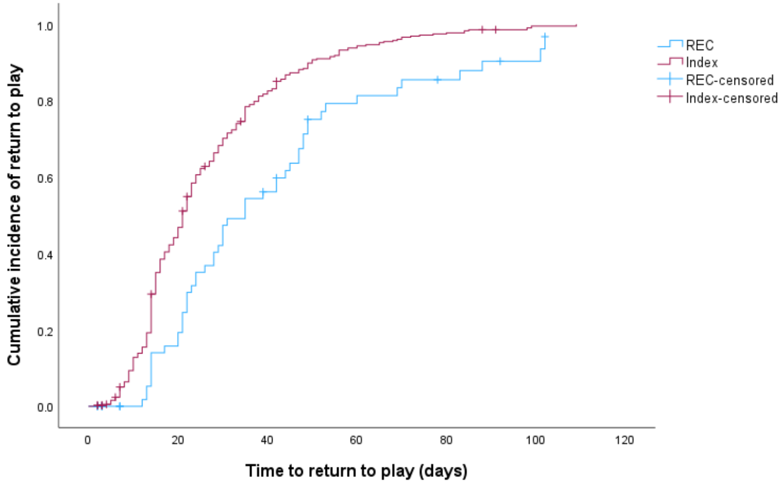

Means and Medians for Survival Time

REC_Vs_Index	Mean				Median			
			95% Confidence Interval				95% Confidence Interval	
	Estimate	Std. Error	Lower Bound	Upper Bound	Estimate	Std. Error	Lower Bound	Upper Bound
REC	42.036	3.649	34.885	49.187	35.000	4.700	25.787	44.213
Index	26.519	.959	24.639	28.400	21.000	.724	19.580	22.420
Overall	28.582	.995	26.632	30.531	22.000	.687	20.654	23.346
